# Schlafen 1 Inhibits the Proliferation and Tube Formation of Endothelial Progenitor Cells

**DOI:** 10.1371/journal.pone.0109711

**Published:** 2014-10-16

**Authors:** Chun-yan Kuang, Tian-he Yang, Yang Zhang, Lu Zhang, Qiang Wu

**Affiliations:** 1 Department of Cardiovascular Diseases, The People’s Hospital of Guizhou Province, Nanming District, Guiyang, People’s Republic of China; 2 Medical examination center, The People’s Hospital of Guizhou Province, Nanming District, Guiyang, People's Republic of China; INSERM, France

## Abstract

Endothelial progenitor cells (EPCs) are the major source of cells that restore the endothelium during reendothelialization. This study was designed to investigate whether Schlafen 1 (Slfn1) has an effect on the proliferation and tube formation of EPCs in vivo. Slfn1 was expressed in rat EPCs. The overexpression of Slfn1 suppressed the proliferation and tube formation of EPCs; conversely, the knockdown of Slfn1 by shRNA promoted the proliferation and tube formation of EPCs. Furthermore, when Slfn1 was overexpressed, the EPCs were arrested in the G1 phase of the cell cycle. In contrast, when Slfn1 was knocked down, the EPCs progressed into the S phase of the cell cycle. Additionally, the overexpression of Slfn1 decreased the expression of Cyclin D1, whereas the knockdown of Slfn1 increased the expression of Cyclin D1; these findings suggest that Cyclin D1 is downstream of Slfn1 in Slfn1-mediated EPC proliferation. Taken together, these results indicate a key role for Slfn1 in the regulation of EPC biological behavior, which may provide a new target for the use of EPCs during reendothelialization.

## Introduction

In the last decade, there have been a number of studies demonstrating that endothelial progenitor cells (EPCs) play a pivotal role in the maintenance of endothelial integrity and hemostasis. EPCs are mobilized from the bone marrow (BM) to sites of damaged endothelium where they differentiate into endothelial cells [1]. Therefore, EPCs have become a therapeutic target for the prevention of restenosis after vascular injury. Several lines of evidence indicate that EPCs are an important marker of cardiovascular diseases, such as hypertension, heart failure, diabetes, and coronary artery disease (CAD), and that EPC number and function are reduced in these diseases [2], [3]. It has become increasingly apparent that many factors, such as CCN1, inflammation, STIM1, and oxidative stress, modulate EPC bioactivity [4]. Recent evidence from the completed first-in-man study of the EPC capture stent suggests that the effectiveness of the stent in reducing the incidence of in-stent restenosis and target vessel revascularization remains uncertain [5]. Overall, the exact mechanism of EPC bioactivity remains poorly understood, which restricts its clinical progress.

The Schlafen (Slfn) family of proteins includes 10 members in mice (Slfn1, 1L, 2, 3, 4, 5, 8, 9, 10, and 14) and 5 members in humans (Slfn5, 11, 12, 13, and 14) [6]–[8]. The expression of the Slfn family members in the rat is largely unknown. Our previous study showed that Slfn1 is expressed in the EPCs of the rat [9]. There is evidence that the Slfn family comprises 3 groups of proteins, which are delineated according to the size of the encoded proteins [10]–[13]. These proteins include a common N-terminal (AAA) domain, which has been implicated in GTP/ATP binding [11], [14]. Previous studies have indicated that Slfn family proteins were involved in the regulation of important biological functions in mammals, such as the induction of immune responses and the regulation of cell proliferation [10], [14]. Slfn1 belongs to group 1 (short Slfns). To date, limited studies conducted on determining the role of Slfn1 in the regulation of cellular functions have found that Slfn1 impairs thymocyte development through the inhibition of Cyclin D1 expression [11], [14]. However, very little is known about whether Slfn1 is involved in vascular injury and repair. EPCs are a major cell source for repair after vascular injury. Previously, we reported that Slfn1 is a downstream target of TRPC1, which regulates EPC proliferation [9]. Therefore, based on these experiments, we hypothesized that Slfn1 was involved in the regulation of the biological functions of EPCs.

The aim of this study was to investigate the role of Slfn1 in EPC proliferation and tube formation in vitro. First, we investigated the subcellular localization of Slfn1 in rat BM-derived EPCs. Next, we investigated whether Slfn1 affected the proliferation and tube formation of EPCs in vivo. Finally, we explored the mechanism by which Slfn1 affects EPC function.

## Materials and Methods

### Ethics statement

Animal procedures were authorized by the Care of Experimental Animals Committee of Daping Hospital (approval reference number A5572-01). The research agrees with the Guide for the Use and Care of Laboratory Animals published via the U.S. National Institute of Health (NIH Publication No. 85-23, revised 1996).

### Isolation and characterization of EPCs

The culture and characterization of the EPCs was performed as previously described [4]. Bone marrow (BM) was harvested by flushing the femurs and tibias of Sprague-Dawley (SD) rats (male, 150–180 g). After the BM was harvested, BM-derived mononuclear cells were isolated using density-gradient centrifugation (Lymphoprep 1.083). After three washing steps, the cells were resuspended using low-glucose Dulbecco’s Modified Eagle’s Medium (DMEM) supplemented with 100 IU/mL penicillin, 10% fetal calf serum (FCS, Gibco, Grand Island, NY, USA), 100 mg/mL streptomycin, and 10 ng/mL vascular endothelial growth factor (VEGF). Finally, the cells were seeded onto culture flasks and incubated at 37°C with 5% CO2. The adherent cells were cultured for 5 to 7 days and used in further experiments. To verify the EPC phenotype, the cells were first incubated using acLDL-Dil (10 mg/mL,Biomedical Technologies, Inc, Stoughton, MA, USA) for 4 h, fixed with 4% paraformaldehyde, incubated with FITC-labeled lectin (UEA-1, 10 mg/mL,Bios, Beijing, China) for 1 h, and examined using a confocal laser scanning microscope (LSCM, FV-300, Olympus, Japan). Cells that stained positive for both UEA-1 and acLDL-DiI were determined to be EPCs. In addition, flow cytometry analysis was performed with antibodies against rat CD133, CD45, VEGFR-2, and CD34 and with the corresponding isotype control antibodies (Bios, Beijing, China).

### Immunocytochemistry

Cultured EPCs were fixed for 15 min at room temperature with 4% paraformaldehyde and washed twice with phosphate-buffered saline (PBS) to detect Slfn1 expression. Endogenous peroxidase activity was inactivated by incubating the cells with 1% H_2_O_2_ for 30 min. After washing, the cells were treated with 0.1% Triton X-100 and incubated with a 1∶200 dilution of a rat Slfn1 primary antibody (Santa Cruz, USA) overnight. Then, the samples were incubated with Cy3-conjugated secondary antibodies (1∶500; Zhongshan, Shanghai, China) for 1 h. The fluorescent signal was detected using a LSCM.

### Recombinant adenoviral vectors expressing Slfn1

rSlfn1 (NCBI Reference Sequence: NC_005109.3) cDNA expression constructs were chemically synthesized (Neuron Biotech, China). The rSlfn1 cDNA was first subcloned into a pGS-1 vector, and then 293 cells were infected with the Ad-Slfn1 recombinant adenovirus in a 12-well plate with MetafecteneTM (Biontex) according to the manufacturer's protocol. Finally, the recombinant Ad-Slfn1 was collected 8 days after transfection and amplified in the 293 cells, producing 2 mL of viral stock. The adenoviruses expressed GFP under a separate promoter, which allowed the infection to be verified. All PCR-amplified cloning and fragment junctions were confirmed by DNA sequencing (Sangon, Shanghai, China). An adenovirus encoding a green fluorescent protein (Ad-control) was used as a control. The Ad-Slfn1 and Ad-control viruses were used for functional assays (see below).

### Cell transduction

The rSlfn1 shRNA plasmid (r) (shRNA-Slfn1) and control shRNA (shRNA-control) plasmid were purchased from Qiagen (Germany).

The transfection of shRNAs and Ad-Slfn1 was performed according to the manufacturer’s instructions. EPCs were transduced with shRNA-Slfn1, shRNA-control, Ad-Slfn1, or Ad-control constructs for 48 h before being used in experiments. Uninfected EPCs were used as blank controls.

### Cell proliferation studies

[3H]-thymidine incorporation was used to study DNA synthesis in the EPCs as previously described [9]. First, EPCs were seeded onto 24-well plates. After serum starvation for 24 h, the cells were transfected with shRNA-Slfn1, shRNA-control, Ad-Slfn1, or Ad-control constructs for 48 h. During the final 6–8 h of transfection, 1 µCi of [methyl-3H]-thymidine was added to each well for 8 h. Finally, incorporated [3H]-thymidine was precipitated with 10% trichloroacetic acid and subsequently counted with a liquid scintillation counter. Furthermore, the cell number (6-well plates, 1×10^6^ cells/well as a baseline) was counted at 0, 24, 48, and 72 h after cell transfection. Each count was conducted an average of 3 times, and every data point was counted in triplicate.

### Tube formation assay

Matrigel of the same batch was thawed and added into 24-well plates at 37°C for 1 h to allow solidification. EPCs transfected with shRNA- Slfn1,shRNA-control,Ad-Slfn1 and Ad-control were harvested, resuspended and placed on the matrigel, EPCs with nontransfected as a control. And then incubated at 37°C in an atmosphere of 5% CO2 for 18 h, EPCs tube formation was examined using microscopy, and the total length of the tubelike was measured with Leica Qwin V3.1 software.

### Cell cycle analysis

The cell cycle distribution was determined with flow cytometry. Briefly, after transfection for 48 h, the EPCs were trypsinized and then centrifuged at 1,500 g for 5 min. The cells were subsequently washed with PBS and fixed in 70% ethanol overnight at 4°C. After fixation, the EPCs were incubated in 0.1% sodium citrate containing 0.05 mg of PI and 100 mg/mL RNase for 30 min at room temperature in the dark. Fluorescence was analyzed using a FACS scan flow cytometer (Beckman). The percentage of cells in different phases of the cell cycle was determined using Cell-FIT software.

### RNA extraction and reverse-transcriptase PCR (RT-PCR)

Total RNA was extracted from EPCs using Trizol (Invitrogen, Carlsbad, CA, USA), and then cDNA was synthesized with oligo(dT) and MMLV reverse transcriptase (Toyobo, Japan). cDNA amplification and semi-quantitative PCR were performed using the following primer pairs.Slfn1 forward 5′-CCA GAT GTC TCT GTT GGG AA-3′ and Slfn1 reverse 5′-GCT AAG ACA TGA GGA GCT TG-3′[9], Cyclin D1 forward 5′- TGC TTG GGA AGT TGT GTT GG-3′ and Cyclin D1 reverse 5′-AAT GCC ATC ACG GTC CCT AC-3′[15], and rat β-actin forward 5′-TCA GGT CAT CAC TAT CGG CAA T-3′ and rat β-actin reverse 5′-AAA GAA AGG GTG TAA AAC GCA-3′. All primers, which were salt-free and of the highest purity, were synthesized by Sangon Biotech (Shanghai).

### Western blot analysis

The protein concentrations of cell lysates were investigated using the Bradford method. First, the same mass of total protein was loaded onto each lane and separated by SDS-PAGE on a 10–15% polyacrylamide gel. Second, the proteins were transferred to polyvinylidene difluoride membranes. The membranes were blocked with 5% non-fat milk, and the membrane-bound proteins were probed with primary antibodies against Slfn1, Cyclin D1, and β-actin followed by incubation with horseradish peroxidase (HRP)–conjugated secondary antibodies. The protein bands were visualized with chemiluminescent detection (ECL, Amersham Biosciences Uppsala, Sweden) and quantified using a gel imaging analysis system (GBOX/CHEM.USA).

### Statistical analysis

All data are expressed as the mean±SD. SPSS 17.0 software was used for analysis. The data were analyzed in pairs (test and control) using a t-test, and a P-value of less than 0.05 was regarded as being statistically significant.

## Results

### Slfn1 is expressed in EPCs

After 4–7 days of culture, adherent EPCs were analyzed with a LSCM and flow cytometry. The majority of cells (90.11±0.42%) stained positive for lectin and acLDL-DiI (Figure S1A) and expressed endothelial/stem cell markers, including VEGFR-2 (97.63%), CD34 (53.6%), and CD133 (87.99%), but did not express CD45 (6.32%) (Figure S1B).

**Figure 1:**
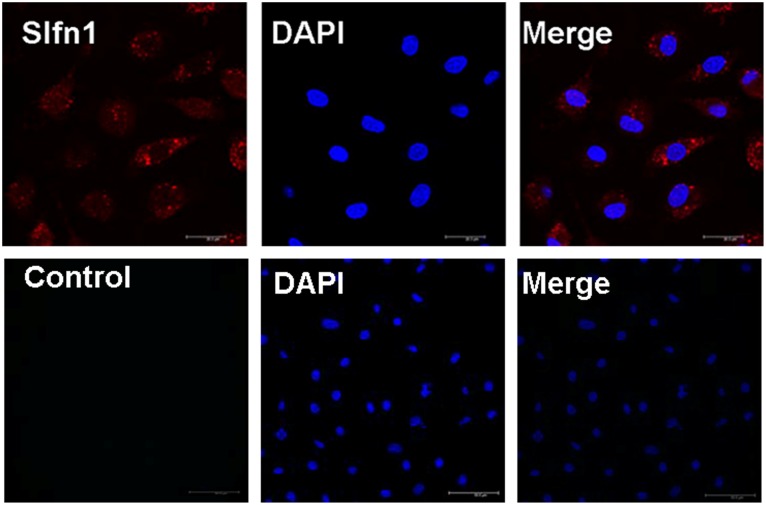
The localization of Slfn1 in primary EPCs. Subcellular localization of Slfn1 in primary EPCs.Top: Primary EPCs were incubated using Cy3-conjugated secondary antibodies and anti-Slfn1 polyclonal primary antibodies. Analysis of the red fluorescent signal (Slfn1) shows that Slfn1 is mainly localized in the cytoplasm with minor localization in the nuclei of EPCs. Bottom: control cells that were not incubated with the anti-Slfn1 antibody did not show green fluorescence.

Our previous study showed that Slfn1 is involved in the regulation of TRPC1 on EPCs [9]. In the present study, we further investigated the subcellular localization of Slfn1 in EPCs with immunocytochemistry. Our results revealed that Slfn1 was predominantly localized in the cytoplasm of EPCs, with minor localization also in the nucleus (Fig. 1). Additionally, control cells that were not incubated with the anti-Slfn1 antibody did not show green fluorescence. Taken together, these observations demonstrated that Slfn1 was expressed in primary EPCs.

### Slfn1 reduced the proliferation and tube formation of EPCs

**Figure 2:**
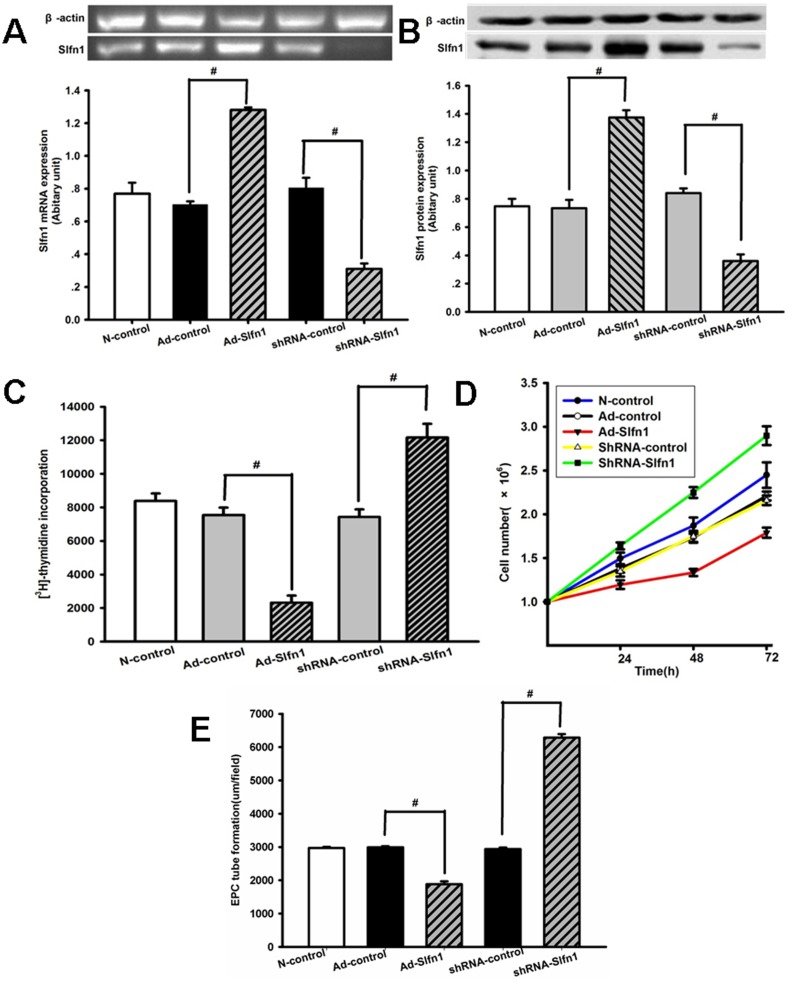
Slfn1 suppresses EPC proliferation and tube formation. A.Slfn1 mRNA expression levels were examined by semi-quantitative RT-PCR. Top: Slfn1 expression was normalized to the expression level of the housekeeping gene *β*-actin. The transduction of EPCs with shRNA-Slfn1 greatly decreased Slfn1 mRNA expression after 48 h, whereas the transduction of EPCs with Ad-Slfn1 clearly increased Slfn1 mRNA expression after 48 h (n = 3). Bottom: Densitometric analysis of Slfn1 mRNA expression levels relative to β-actin were determined by the Quantity One program. The results are expressed as the mean±SEM. #P<0.05. **B.** Slfn1 protein levels were detected using western blot analysis. Top: Slfn1 expression levels normalized to the expression level of the housekeeping gene β-actin. The transduction of EPCs with shRNA-Slfn1 clearly decreased Slfn1 protein expression after 48 h. Conversely, the transduction of EPCs with Ad-Slfn1 greatly increased Slfn1 protein expression after 48 h (n = 3). Bottom: Densitometric analysis of Slfn1 protein expression levels relative to β-actin were determined by the Quantity One program. The results are expressed as the mean±SEM. ^#^P<0.05. **C.** A [3H]-thymidine incorporation assay was used to investigate EPC proliferation. The transfection of EPCs with Ad-Slfn1 clearly decreased the uptake of [3H]-thymidine by EPCs after 48 h. Conversely, the transfection of EPCs with shRNA-Slfn1 increased the uptake of [3H]-thymidine by EPCs after 48 h. The data are presented as the mean±SD (n = 3). ^#^P<0.05. **D.** A cell count was used to measure EPC proliferation. The transfection of EPCs using Ad-Slfn1 significantly inhibited the proliferation of EPCs after 48 h. Conversely, the transfection of EPCs with shRNA-Slfn1 clearly increased the proliferation of EPCs after 48 h. Values are presented as the mean±SD (n = 3), ^#^P<0.05. **E.** Tube formation by EPCs transfected with shRNA- Slfn1, shRNA-control, Ad-Slfn1 and Ad-control were performed as described in Materials and methods, EPCs images were captured and measured by Leica Qwin system as depicted in the legends to Fig. 2E. The data are shown as the mean±S.D. of total length per field ((n = 3),^ #^P<0.05.

To evaluate the effects of Slfn1 on EPCs. The proliferation of rat BM- derived EPCs was investigated. shRNA-Slfn1, shRNA-control, and adenovirus constructs expressing Ad-control and Ad-Slfn1 were transfected into the EPCs. After 48 h, the transfection efficiency of the adenovirus and the shRNA constructs as calculated by GFP expression was 80.3%±1.5% (n = 3, Ad-control and Ad-Slfn1, Figure S2) and 85.1%±1.42% (n = 3, shRNA-control and shRNA-Slfn1, Figure S3) respectively. The Slfn1 expression levels were determined by western blotting and semi-quantitative RT-PCR 48 h after transduction. Transfection with Ad-Slfn1 significantly increased Slfn1 protein and mRNA expression compared with the controls (protein levels: 1.3745±0.0518 vs. 0.7338±0.0583, respectively, n = 3, P<0.05, Fig. 2B and mRNA levels: 1.2804±0.0166 vs. 0.7035±0.0201, respectively, n = 3, P<0.05, Fig. 2A). However, transfection with shRNA-Slfn1 significantly attenuated Slfn1 mRNA expression compared with the shRNA-control (0.3108±0.0327 from 0.8054±0.0612, respectively, n = 3, P<0.05, Fig. 2A), whereas the Slfn1 protein levels decreased from 0.8399±0.0334 in the shRNA-control cells to 0.3600±0.0472 in the shRNA-Slfn1 transfected cells (n = 3, P<0.05, Fig. 2B). These results show that transfection with Ad-Slfn1 and shRNA-Slfn1 was effective in rat EPCs.

First, We used a [3H]-thymidine incorporation assay to assess the effects of Slfn1 on EPC proliferation. The results show that the transfection of EPCs with Ad-Slfn1 decreased the uptake of [3H]-thymidine post-infection compared with the transfection of the Ad-control (2324.60±413.30 vs. 7532.90±447.22, n = 9, P<0.05, Fig. 2C). However, the transfection of EPCs with shRNA-Slfn1 increased the uptake of [3H]-thymidine post-infection when compared with shRNA–control transfection (7431.70±445.06 vs. 12156.00±819.92, n = 9, P<0.05, Fig. 2C).

In addition, a cell count was completed to further study the effects of Slfn1 on EPC proliferation.Transfection with Ad-Slfn1 inhibited EPC proliferation compared with Ad-control transfection (n = 3, P<0.05, Fig. 2D). In contrast, transfection with shRNA-Slfn1 significantly increased EPC proliferation compared with shRNA -control transfection (n = 3, P<0.05, Fig. 2D).

Lastly, to investigate whether Slfn1 affects the ability of EPCs to form capillary- like tubes, matrigel angiogenesis assay was performed with EPCs. The total length of capillary- like tubes structures after 18 h culture were examined.These results indicated that overexpresion of Slfn1 significantly decreased tube formation of EPCs compared with Ad-control transfection (n = 3, P<0.05, Fig. 2E),conversely, transfection with shRNA-Slfn1 significantly increased tube formation of EPCs compared with shRNA-control transfection (n = 3, P<0.05, Fig. 2E),

Taken together, all these results demonstrated that Slfn1 reduced the proliferation and tube formation of EPCs.

### Cyclin D1 is a downstream target of Slfn1

**Figure 3:**
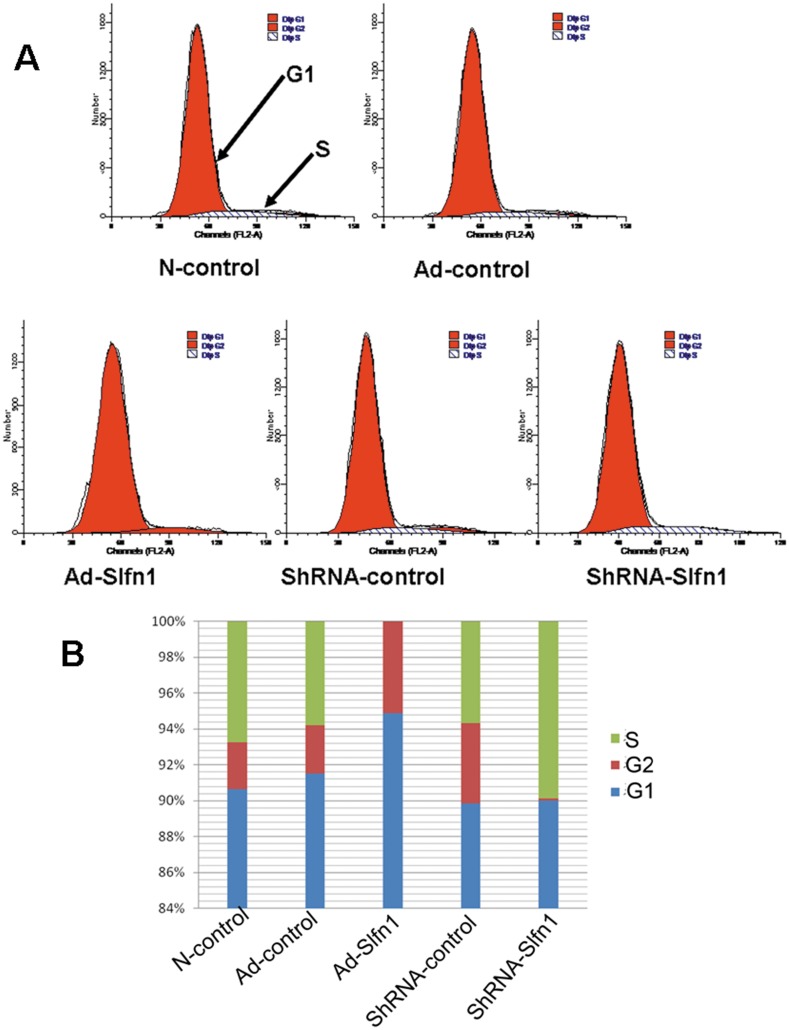
The effect of Slfn1 on the cell cycle regulation of EPCs. Flow cytometry analysis was conducted on EPCs transfected with shRNA-Slfn1, shRNA-Slfn1, Ad-control, or Ad-Slfn1 constructs for 48 h. **A.** A representative pattern of the cell cycle distribution of EPCs in synchrony and after transfection. The transfection of EPCs with Ad-Slfn1 decreased the number of cells in the S phase and increased the number of cell in the G1 phase, whereas the knockdown of Slfn1 with shRNA increased the number of cells in the S phase and decreased the number of cells in the G1 phase. **B.** Average cell cycle distribution data from three different experiments.

To investigate the mechanism by which Slfn1 affects EPC proliferation, the cell cycle phase distribution was assessed using flow cytometry 48 h after the overexpression or silencing of Slfn1. First, EPCs were serum starved for 24 h to obtain synchronization in G0. Next, synchronized cells were transfected with Ad-Slfn1, Ad-control, shRNA-Slfn1, or shRNA-control for 48 h. The cell cycle phase distribution was determined using FACS. Our results show that 0% of EPCs infected with the Ad-Slfn1 construct progressed into the S phase (n  = 3, Fig 3. A.B) and that EPCs infected with Ad-Slfn1 were mainly in the G1 phase (94.88%, n = 3, Fig 3. A.B). However, 9.89% of EPCs infected with the shRNA-Slfn1 construct progressed into the S phase (n = 3, Fig 3. A.B). These results indicate that Slfn1 inhibited EPC proliferation by causing an arrest in the G1 phase of the cell cycle.

**Figure 4:**
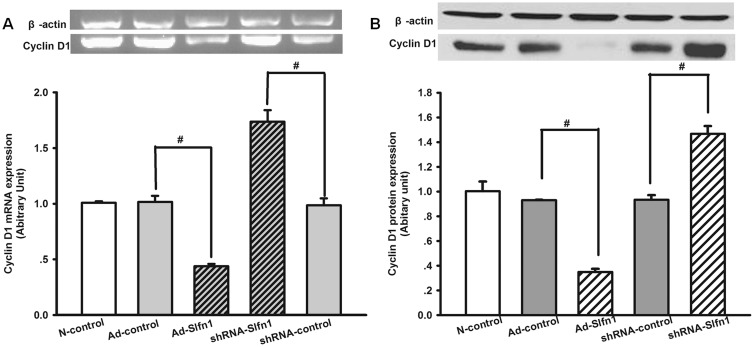
The effect of Cyclin D1, a downstream target of Slfn1, on EPCs. **A.** Slfn1 mRNA levels were measured by semi-quantitative RT-PCR. Top: Slfn1 expression level normalized to the expression level of the housekeeping gene β-actin. The transduction of EPCs with shRNA-Slfn1 clearly increased Cyclin D1 mRNA expression after 48 h, whereas the transduction of EPCs with Ad-Slfn1 greatly decreased Cyclin D1 mRNA expression after 48 h (n = 3). Bottom: Densitometric analysis of Cyclin D1 mRNA expression levels relative to β-actin were determined by the Quantity One program. The results are expressed as the mean±SEM. ^#^P<0.05. **B.** Cyclin D1 protein levels were measured by western blot analysis. Top: Cyclin D1 expression levels normalized to the expression levels of the housekeeping gene β-actin. The transduction of EPCs with shRNA-Slfn1 significantly increased Cyclin D1 protein expression after 48 h. Conversely, the transduction of EPCs with Ad-Slfn1 clearly decreased Cyclin D1 protein expression after 48 h (n = 3). Bottom: Densitometric analysis of the Cyclin D1 protein expression levels relative to β-actin were determined by the Quantity One program. The results are expressed as the mean±SEM. ^#^P<0.05.

A previous study showed that Cyclin D1 is downstream of Slfn1 [14]. To further evaluate if Cyclin D1 is involved in the Slfn1 regulation of EPC proliferation, Cyclin D1 levels were analyzed by western blotting and semi-quantitative RT-PCR 48 h after transduction. As illustrated in Fig. 4A and B, transfection with Ad-Slfn1 significantly decreased Cyclin D1 protein and mRNA expression compared with the Ad-controls (mRNA levels: 0.4388±0.02 vs. 1.012±0.05, n = 3, P<0.05; protein levels: 0.35±0.03 vs. 0.9311±0.00, n = 3, P<0.05). Conversely, transfection with shRNA-Slfn1 significantly increased Cyclin D1 protein and mRNA expression compared with the shRNA–controls (mRNA levels: 1.74±0.11 vs. 0. 99±0.06, n = 3, P<0.05; protein levels: 0.93±0.03 vs. 1.47±0.06, n = 3, P<0.05). Taken together, these data indicate that Slfn1 regulates EPC proliferation via Cyclin D1.

## Discussion

The major findings of the present study are as follows: (1) Slfn1 is dynamically located in the cytoplasm of EPCs in the rat and only partly located in the nucleus. (2) Slfn1 regulates the biological behavior of EPCs. For example, the overexpression of Slfn1 inhibited the proliferation and tube formation of EPCs, whereas the knockdown of Slfn1 by shRNA promoted the proliferation and tube formation of EPCs. (3) When Slfn1 is overexpressed, EPCs are arrested in the G1 phase of the cell cycle.conversely, when Slfn1 is knocked down with shRNA, EPCs progress into the S phase of the cell cycle. (4) Cyclin D1 is downstream of Slfn1 in Slfn1-mediated EPC proliferation and tube formation. Taken together, these observations demonstrate that Slfn1 is a powerful negative regulator of EPC proliferation and tube formation in vitro.

Slfn1 was originally identified in the mouse in 1998. Since then, there has been emerging evidence that indicates that Slfn1 is expressed in both mice and humans [6], [14]. Schwarz et al. reported that Slfn1 is dynamically expressed in the lymph node, thymus, and spleen and at low levels in the lungs [14]. To date, whether Slfn1 is expressed in the heart and vasculature remains unknown. In the present study, we established that Slfn1 is expressed in rat EPCs and that this expression is primarily localized in the cytoplasm, with minor localization in the nucleus, which is consistent with the study by Neumann et al [7]. Our data suggest that Slfn1 is expressed in rat EPCs.

Although little is known about the function of Slfn1, there is evidence suggests that Slfn1 inhibits T-lymphocyte growth [14]. The function of Slfn1 in most cells is not yet known, subsequent studies revealed that Slfn1 is involved in the regulation of TRPC1 in EPC proliferation [9]. In the present study, our results show that Slfn1 plays an essential role in the control of EPC proliferation and tube formation. The overexpression of Slfn1 resulted in the suppression of EPC proliferation and tube formation, whereas the knockdown of Slfn1 using shRNA led to an increase in EPC proliferation and tube formation. Our results indicate that Slfn1 is an important factor in controlling EPC biological behavior. However, there are three main cell types–EPCs, smooth muscle cells, and endothelial cells–that are involved in the processes of vascular injury and repair. Thus, more research is needed to determine whether Slfn1 also regulates the biological behavior of smooth muscle cells and endothelial cells.

It is now well established that Slfn1 controls cell growth by regulating the expression of Cyclin D1. Overexpression of Slfn1 reduces the activation of the cyclin D1 and inhibits fibroblasts cell growth, but overexpression of cyclin D1 in Slfn1 expressing fibroblasts cells with growth-arrested resulted in an increase in cell growth [11]. Notably, a mechanism for the induction of Cyclin D1 via Slfn1 has been described. DnaJB6 enhances Slfn1 nuclear localization, which downregulates Cyclin D1 and ultimately induces cell-cycle arrest [16]. Interestingly, our study also showed that the overexpression of Slfn1 decreases the expression of Cyclin D1, a positive cell cycle regulator, which further confirmed that Slfn1 functions as a negative regulator of cell growth. The knockdown of Slfn1 using shRNA increased the expression of Cyclin D1. Cyclin D1 regulates cell proliferation via the control of cell-cycle progression [17]. Based on this observation, we focused on the Slfn1-mediated regulation of EPC cell-cycle progression. Our results demonstrate that the overexpression of Slfn1 in EPCs induced cell cycle arrest in the G1 phase of the cell cycle. In contrast, the knockdown of Slfn1 induced the progression of EPCs to S phase of the cell cycle. Therefore, our results revealed that Slfn1 inhibits the proliferation of EPCs via its downstream target Cyclin D1, which regulates EPC proliferation by controlling cell-cycle progression. In addition, IFNa and TRPC1, molecules upstream of Slfn1, were studied. IFNa obviously induced the mRNA expression of Slfn1 in mouse embryonic fibroblast cell lines, and Stat1 and p38 MAPK were found to be required for induction of the IFN-inducible mouse Slfn1 [18]. Our previous study also suggest that TRPC1 regulates EPC proliferation through its downstream target Slfn1 [9].

The tube formation starts with the proliferation and migration of EPCs while many factors affecting the proliferation and migration of EPCs will have an effect on the process of the tube formation. Our study discovered that overexpression of Slfn1 leads to the suppression of EPC proliferation, migration and tube formation via the regulation of Cyclin D1,this agrees with the results in [19]–[21] which reported that down-regulate Cyclin D1 expression can suppress tumor and human umbilical vein endothelial cell angiogenesis. This indicates that Slfn1 modulates EPC tube formation by Cyclin D1 regulating EPC proliferation. In addition**,** the crucial signal of tube formation is the release of vascular endothelial growth factor (VEGF)[22]. Cyclin D1 silencing results in a decrease in VEGF expression in DLD1, and Cyclin D1 significantly inhibits in vitro tube formation in VEGF-treated human umbilical vein endothelial cells [23]. We see that additional studies of VEGF involving in Cyclin D1 in EPCs would help to understand deeply how Slfn1 regulates EPC tube formation via Cyclin D1, and this will be our future work.

To date, the function of Slfn1 has been investigated only to a limited degree although the gene is not new. Further research on detailed function of the gene is needed. This will help understand the behaviour and the role of Slfn1 in more cells and organs.

In summary, our findings indicate a key role for Slfn1 in the regulation of EPC biological behavior, which may provide a novel target for the use of EPCs during repair after vascular injury.

## Supporting Information

Figure S1 **Characteristics of bone marrow–derived endothelial progenitor cells (EPCs). A.** EPCs stained positive for lectin (green) and acetylated LDL (red) (90.11±0.42%, *n* = 3; three random fields per well). **B.** Flow cytometry analysis of primary EPCs cultured for 7 days. EPCs labeled with fluorescent antibodies recognizing VEGFR-2, CD133, CD45, and CD34 are exhibited as light green areas. The corresponding negative controls are shown as the gray areas in each box, the lines represent the positive gate, and the numbers indicate the percentage of positive cells.
(TIF)

Figure S2 **The transfection efficiency of Ad-GFP.** The transfection efficiency for adenovirus GFP vectors (cells fluorescing/total number of cells) in cultured EPCs was 80.3%±1.5%.
(TIF)

Figure S3 **The transfection efficiency of ShRNA-GFP.** The transfection efficiency for GFP vectors (cells fluorescing/total number of cells) in cultured EPCs was 85.1%±1.42%.
(TIF)
