# Supplementary figures and images for: Schlafen 1 Inhibits the Proliferation and Tube Formation of Endothelial Progenitor Cells
Source: PLoS One. 2014 Oct 16;9(10):e109711. doi: 10.1371/journal.pone.0109711 (PMC4199616; doi:10.1371/journal.pone.0109711)

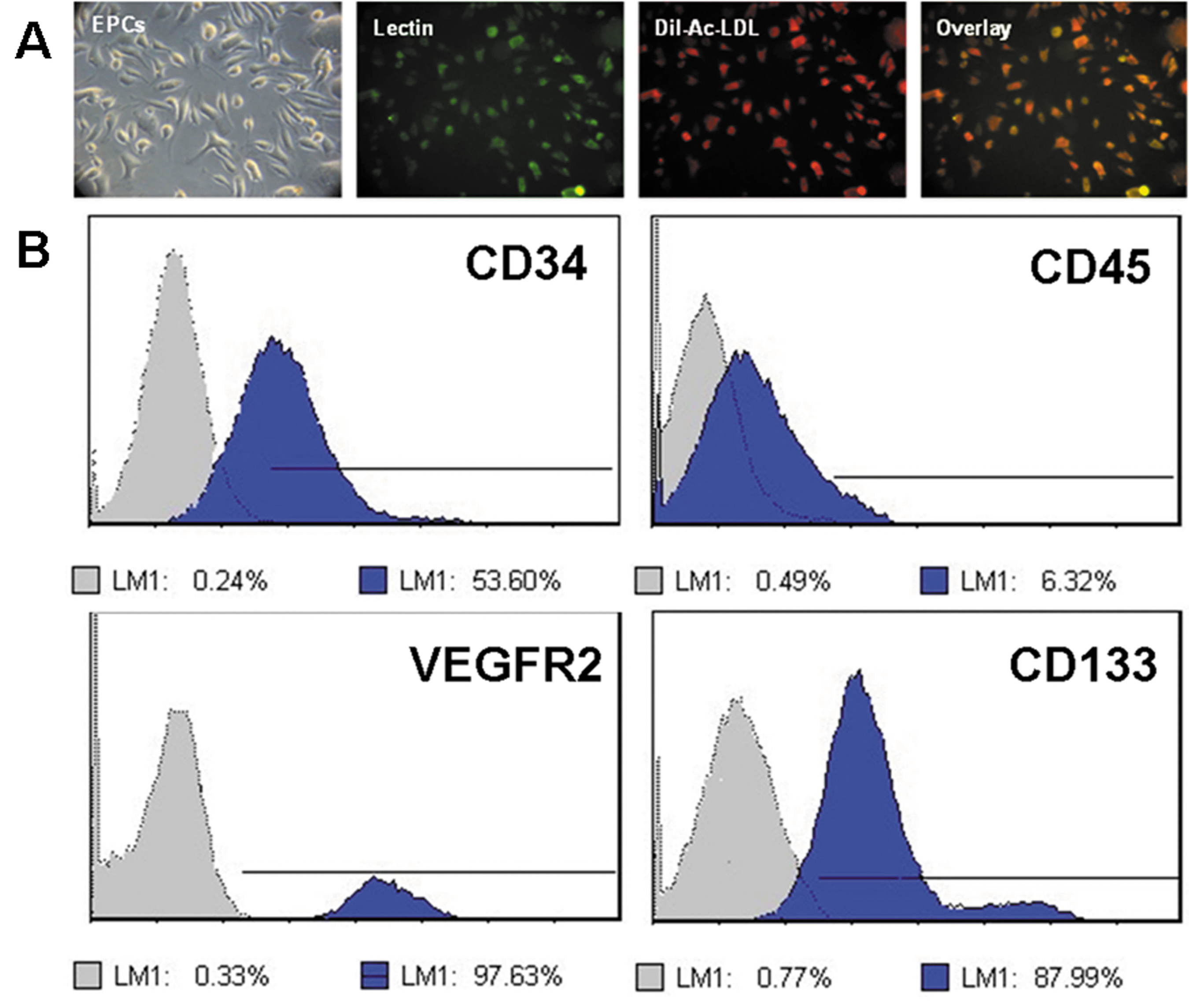

Supplement: Figure S1 — Characteristics of bone marrow–derived endothelial progenitor cells (EPCs). A. EPCs stained positive for lectin (green) and acetylated LDL (red) (90.11±0.42%, n = 3; three random fields per well). B. Flow cytometry analysis of primary EPCs cultured for 7 days. EPCs labeled with fluorescent antibodies recognizing VEGFR-2, CD133, CD45, and CD34 are exhibited as light green areas. The corresponding negative controls are shown as the gray areas in each box, the lines represent the positive gate, and the numbers indicate the percentage of positive cells. (TIF) [file pone.0109711.s001.tif]

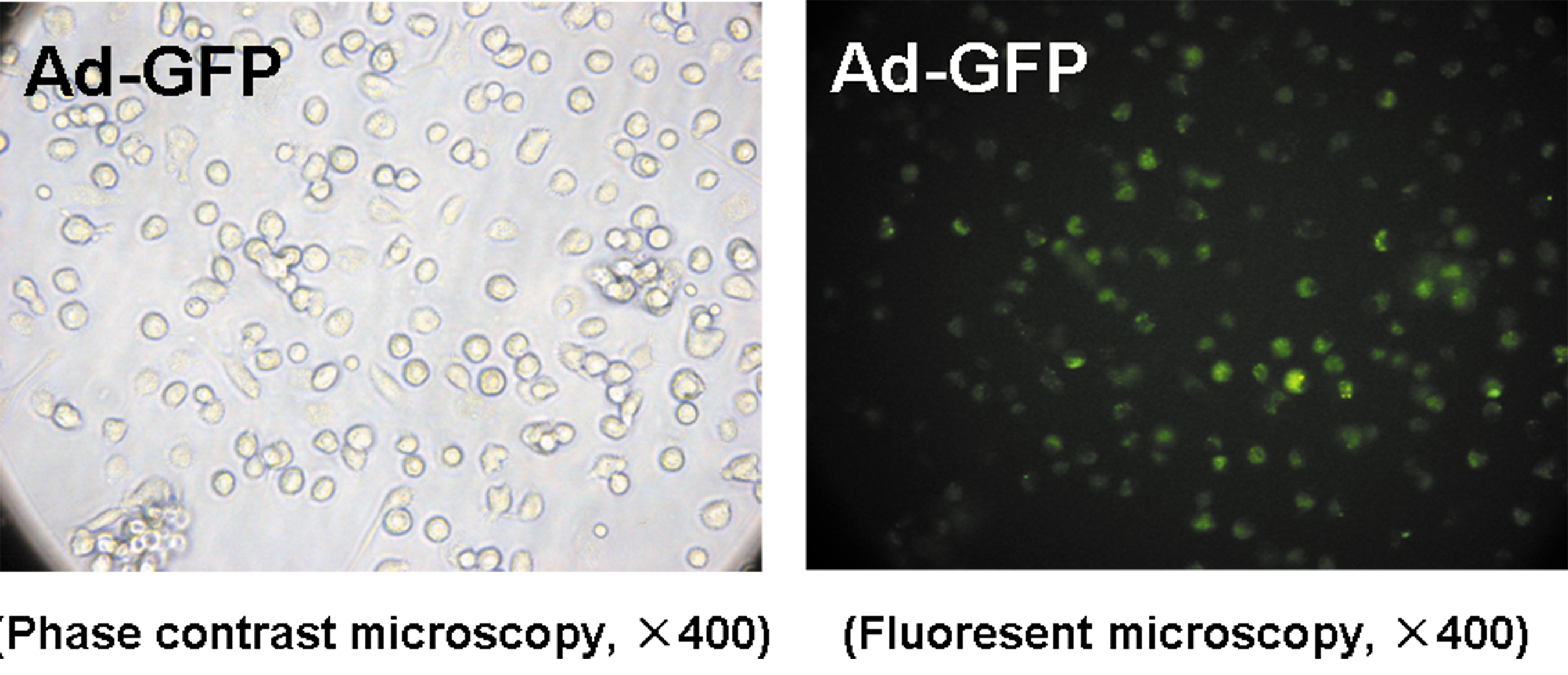

Supplement: Figure S2 — The transfection efficiency of Ad-GFP. The transfection efficiency for adenovirus GFP vectors (cells fluorescing/total number of cells) in cultured EPCs was 80.3%±1.5%. (TIF) [file pone.0109711.s002.tif]

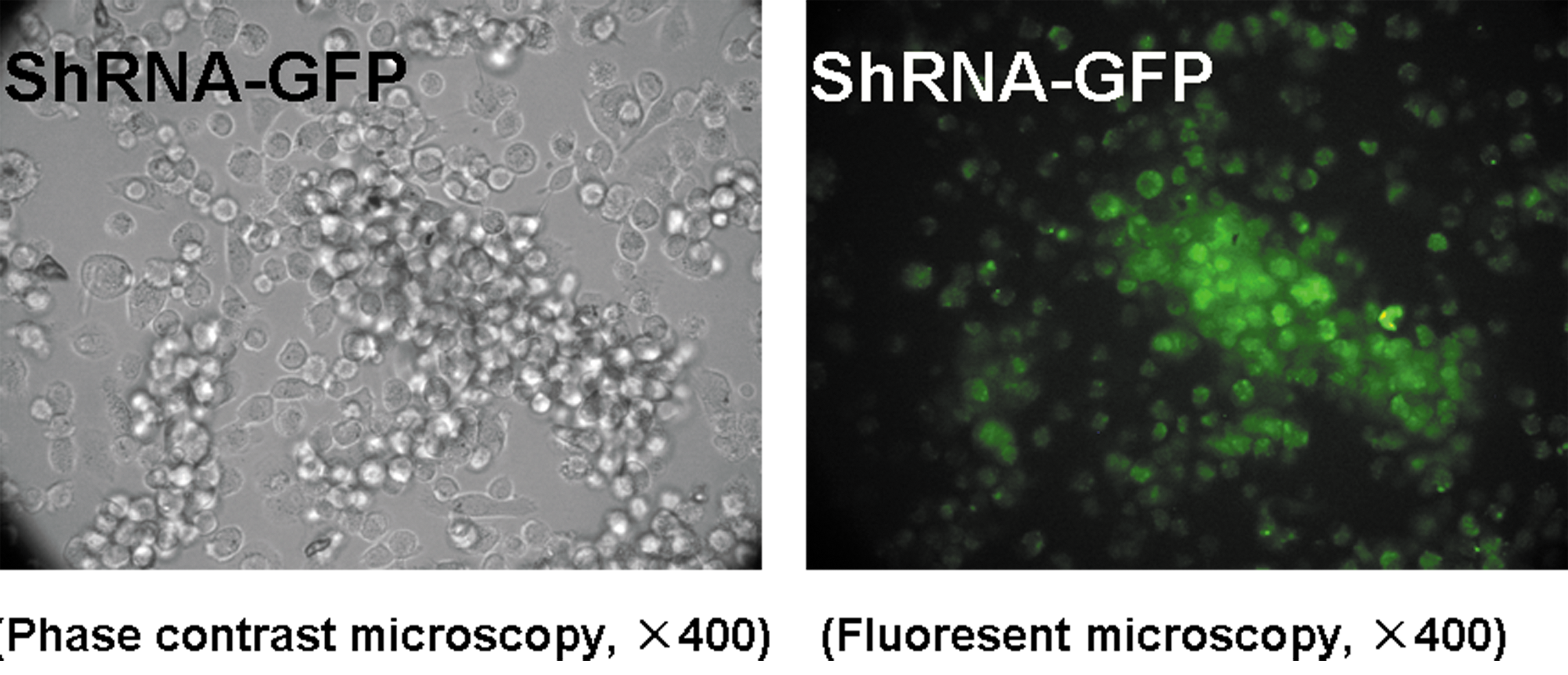

Supplement: Figure S3 — The transfection efficiency of ShRNA-GFP. The transfection efficiency for GFP vectors (cells fluorescing/total number of cells) in cultured EPCs was 85.1%±1.42%. (TIF) [file pone.0109711.s003.tif]
